# From Anatomy to Genomics Using a Multi-Task Deep Learning Approach for Comprehensive Glioma Profiling

**DOI:** 10.3390/bioengineering12090979

**Published:** 2025-09-15

**Authors:** Akmalbek Abdusalomov, Sabina Umirzakova, Obidjon Bekmirzaev, Adilbek Dauletov, Abror Buriboev, Alpamis Kutlimuratov, Akhram Nishanov, Rashid Nasimov, Ryumduck Oh

**Affiliations:** 1 Department of Computer Engineering, Gachon University Sujeong-Gu, Seongnam-Si 13120, Republic of Korea; akmaljon@gachon.ac.kr (A.A.); sabinatuit@gachon.ac.kr (S.U.); 2 Department of Information Processing and Management Systems, Tashkent State Technical University, Tashkent 100095, Uzbekistan; 3 Department of Artificial Intelligence, Tashkent State University of Economics, Tashkent 100066, Uzbekistan; bekmirzaev_obidjon@tsue.uz (O.B.); rashid.nasimov@tsue.uz (R.N.); 4 Department of Digital Technologies, Alfraganus University, Tashkent 100190, Uzbekistan; a.dauletov@afu.uz; 5 Department of AI-Software, Gachon University, Sujeong-Gu, Seongnam-Si 13120, Republic of Korea; abror1989@gachon.ac.kr; 6 Department of Applied Informatics, Kimyo International University, Tashkent 100170, Uzbekistan; kutlimuratov.aj@kiut.uz; 7 Faculty of Software Engineering, Tashkent University of Information Technologies Named After Muhammad Al-Khwarizmi, Tashkent 100200, Uzbekistan; a.nishanov@tuit.uz; 8 Department of Computer Systems, Tashkent University of Information Technologies Named After Muhammad Al-Khwarizmi, Tashkent 100200, Uzbekistan; 9 Department of Computer Software, Korea National University of Transportation, Chungju-si 27909, Republic of Korea

**Keywords:** integrated glioma diagnosis, multi-task medical imaging, volumetric brain MRI analysis, non-invasive molecular profiling, end-to-end tumor characterization

## Abstract

Background: Gliomas are among the most complex and lethal primary brain tumors, necessitating precise evaluation of both anatomical subregions and molecular alterations for effective clinical management. Methods: To find a solution to the disconnected nature of current bioimage analysis pipelines, where anatomical segmentation based on MRI and molecular biomarker prediction are done as separate tasks, we use here Molecular-Genomic and Multi-Task (MGMT-Net), a one deep learning scheme that carries out the task of the multi-modal MRI data without any conversion. MGMT-Net incorporates a novel Cross-Modality Attention Fusion (CMAF) module that dynamically integrates diverse imaging sequences and pairs them with a hybrid Transformer–Convolutional Neural Network (CNN) encoder to capture both global context and local anatomical detail. This architecture supports dual-task decoders, enabling concurrent voxel-wise tumor delineation and subject-level classification of key genomic markers, including the IDH gene mutation, the 1p/19q co-deletion, and the TERT gene promoter mutation. Results: Extensive validation on the Brain Tumor Segmentation (BraTS 2024) dataset and the combined Cancer Genome Atlas/Erasmus Glioma Database (TCGA/EGD) datasets demonstrated high segmentation accuracy and robust biomarker classification performance, with strong generalizability across external institutional cohorts. Ablation studies further confirmed the importance of each architectural component in achieving overall robustness. Conclusions: MGMT-Net presents a scalable and clinically relevant solution that bridges radiological imaging and genomic insights, potentially reducing diagnostic latency and enhancing precision in neuro-oncology decision-making. By integrating spatial and genetic analysis within a single model, this work represents a significant step toward comprehensive, AI-driven glioma assessment.

## 1. Introduction

Gliomas are the most frequent malignant primary brain tumors in adults, representing a high percentage of the central nervous system neoplasms [1]. In both their anatomical presentation and molecular composition they have been found to display excessive heterogeneity, thus becoming very difficult in the case of accurate diagnosis and treatment planning [2]. The correct performance of clinical therapies relies greatly on the exact identification of the extent of the tumor as well as the accurate recognition of the main molecular biomarkers like the isocitrate dehydrogenase (IDH) mutation, 1p/19q co-deletion, and telomerase reverse transcriptase (TERT) promoter mutation [3]. These biomarkers are core elements of the World Health Organization (WHO) classification of central nervous system tumors and have a great impact on the prognosis and therapy; therefore, the right surgical approach, radiotherapy protocol, and targeted drug treatment are the ones that would be selected [4]. Nevertheless, the present diagnostic pipelines still segregate the tumor anatomical segmentation from molecular profiling as two separate, independent tasks, although the advancements in neuro-oncological research are considerable [5]. Such a separation not only causes redundant data processing, increases computational overhead, and decreases clinical integration [6], but also fails to recognize the spatial–genomic interplay in which Gliomas are inherent [7].

Human brain scans by Magnetic Resonance Imaging (MRI) continue to be the leading method for non-invasive diagnosis of glioma because of their outstanding capability to differentiate soft tissues and the multi-planar imaging feature [8]. Usually, MRI is performed in multiple sequences, each sequence representing a certain acquisition protocol aimed at making a certain tissue characteristic more evident [9]. As for glioma diagnostics, the four most significant sequences are T1-weighted (T1), contrast-enhanced T1-weighted (T1_ce_), T2-weighted (T2), and Fluid-Attenuated Inversion Recovery (FLAIR) [10]. T1_ce_ allows for the clear visualization of the actively enhancing tumor areas and neovascularization, T2 discloses the presence of edema and necrosis, and FLAIR is quite sensitive in showing the infiltrative tumor margins as well as the peritumoral swelling [11]. Anatomical segmentation as well as molecular marker prediction therapies are heavily reliant on this multi-sequence data [12]. In addition, different MRI sequences give different pieces of the diagnostic puzzle that complement each other, and their relative use can be different in different regions of the tumor depending on the pathology and microenvironment [13].

Generally, although there have been big steps in both the imaging and the computational parts [14], routine clinical and computational methods still treat tumor segmentation and molecular prediction as separate processes [15]. In most cases, voxel-wise segmentation is carried out by U-Net-based convolutional neural networks (CNNs) or similar structures [16]. On the other hand, molecular classification is considered a different problem that is solved by using manually extracted radiomic features or by using embeddings learned from volumetric data [17]. Such a separate manner of working brings inefficiencies, model complexity gets bigger, and the clinical scalability is lower [18]. More importantly, it does not allow making use of the diagnostic relationships between tumor imaging phenotypes and the changes in the genome, whereby mutations occurring in certain regions are most times closely linked with specific radiological features.

Recent deep learning technology has successfully demonstrated the advantages of Vision Transformers and 3D CNNs to completely understand the context of a medical image and include the necessary local anatomical detail [19]. Nevertheless, most of the current models just use one single modality without taking into consideration hybrid architectures for multi-task learning. On the other hand, multi-modal MRI fusion methods for brain tumor diagnosis are usually limited to simple channel stacking or static weighting that do not consider the dynamic and spatially varying importance of different modalities. Although channel attention blocks and squeeze-and-excitation (SE) mechanisms have provided some degree of flexibility [20], these methods mostly function at a global, coarse level without voxel-level accuracy. The newly developed cross-modal attention techniques allow for the area-specific adaptive integration of modalities; however, they are still largely underutilized in fully end-to-end multi-task systems for neuro-oncology.

To address these gaps, we are presenting MGMT-Net, an innovative, all-inclusive deep learning framework for detailed glioma identification from multi-modal 3D MRI. We have redefined the diagnostic process by linking non-invasive molecular biomarker prediction and volumetric tumor segmentation into a single architecture that is MGMT-Net:

- Cross-Modality Attention Fusion (CMAF)—a spatially adaptive fusion module that flexibly identifies the voxel-level significance of different MRI modalities, thus highlighting the most diagnostically valuable features in different tumor regions.
- Hybrid Transformer–CNN Encoder—effectively melding 3D CNNs’ local texture sensitivity with 3D Swin Transformers (Shifted Window Transformer) long-range dependency modeling to both fine anatomical detail and broader structural context.
- Dual Task-Specific Decoders—allowing separate yet synergistic celibacy for voxel-wise tumor segmentation and subject-level genomic classification, thus preventing inter-task interference while harnessing shared representations.

We assume that MGMT-Net would be able to beat single-task models that are based on space and genome, and that it would realize higher accuracy, efficiency, and generalizability. We have implemented the framework on the BraTS 2024 dataset [21] for segmentation, on the TCGA/Erasmus Glioma Database (EGD) [22] for molecular prediction, as well as on multi-institutional external cohorts. Our findings depict top-notch performance in both tasks, along with remarkable stability across different institutions, thus opening the door for MGMT-Net in clinical neuro-oncology.

## 2. Related Works

Gliomas pose difficult challenges for precise diagnosis, treatment planning, and prognostication, as they are one of the most heterogeneous and aggressive primary brain cancers. The evolution of multi-modal magnetic resonance imaging (MRI) has provided the capacity for rich, non-invasive imaging and characterization of these tumors in an anatomical and physiological sense. At the same time, the emergence of deep learning in artificial intelligence has transformed imaging in neuro-oncology by providing tumor segmentation and molecular marker prediction capabilities through deep data-driven systems. However, most models do not capture the full presentation complexity of gliomas. They perform the tasks in disregard of each other, or they neglect spatial adaptation in multi-modal fusion. It is based on poorly adapted architecture with insufficient spatial adaptivity. To frame my proposed approach within the existing body of research, we will summarize the latest advances in four key areas that directly inform our work: (1) brain tumor segmentation using deep learning; (2) molecular biomarker assessment from MRI; (3) multi-task learning in neuro-oncology. While progress has been made in all areas, there is a lack of a cohesive, modality-autonomous, and task-aware framework governing them.

### 2.1. Brain Tumor Segmentation

Segmenting glioma subregions from multi-modal MRI scans is fundamental to neuro-oncological imaging, yet remains challenging due to the heterogeneous shape and appearance of gliomas. Fully convolutional networks (FCNs), specifically the U-Net and its 3D versions, have dominated this area, performing segmentation. These models included encoder–decoder structures with skip connections designed to preserve spatial detail [23]. Segmentation accuracy was further improved by the adaptations Residual U-Net, Attention U-Net [24], and self-configuring nnU-Net, which incorporated residual learning, attention mechanisms, and automated hyperparameter tuning, respectively [25]. These convolution-based approaches are especially proficient at modeling local spatial features. They completely fail to capture the wide-ranging dependencies that are characteristic of the diffuse and intricate anatomy of gliomas. To relax these constraints, more recent research has applied Vision Transformers, which model global context via self-attention mechanisms [26]. Swin Transformers have proved particularly adept in volumetric contexts because they compute attention within shifted windows, thus providing scalable global information flow [27,28]. Transformer-based architectures have enhanced contextual reasoning; however, they are expensive in terms of computational resources and often need large-scale datasets. This development has generated interest in investigating new hybrid architectures that try to integrate the locality-sensitivity of CNNs with the global modeling capabilities of Transformers.

### 2.2. Molecular Marker Prediction from MRI

At the same time as anatomical segmentation, the prediction of molecular biomarkers for gliomas—like IDH mutation, 1p/19q co-deletion, and TERT promoter mutation—has become increasingly important in the context of radiogenomics. These biomarkers form an integral part of the glioma WHO classification system and significantly impact the prognosis and treatment strategy [29]. In this domain, earlier computational methods utilized a combination of handcrafted radiomic features and machine learning classifiers, including random forests and support vector machines. While these approaches were interpretable, they were limited by reliance on manual feature extraction and an inability to capture nonlinear relationships between imaging and genomics [30]. In more recent work, deep neural networks, namely 3D CNNs and DenseNets, have been used to learn latent features from volumetric MRI scans directly [31]. Though these models have been successful, they often approach molecular prediction as a global classification problem, treating it as spatially agnostic segmentation. Consequently, they miss the spatial context that is crucial to many imaging-genomic relationships—for example, necrotic cores associated with IDH-mutant gliomas. To improve interpretability and focus, attention-based pooling and class activation maps (CAMs) are used. However, their use in multi-modal, multi-task frameworks remains sparse.

### 2.3. Multi-Task Learning in Neuro-Oncology

Multi-task learning (MTL) conveniently offers a method to simultaneously integrate tumor segmentation with molecular marker forecasting by leveraging common shared feature representations to boost generalization and mitigate overfitting [32]. In neuro-oncology, some works have focused on dual-task systems aimed at concurrent spatial and deep semantic reasoning—for instance, grading or classifying the type of tumor [33]. These models usually employ shared encoders with task-specific decoders, but there is often no satisfactory solution for multi-modal fusion and inter-task interference. Most common MTL implementations, like MTNets and multi-branch networks, tend to merge input modalities through basic concatenation, treating all features uniformly across tasks [34]. This reasoning fails because different sequences of MRI scans have spatial- and subtype-dependent diagnostic value for specific molecular functions, which heavily limits performance and interpretability. Multi-modal fusion is still one of the most important and least developed features concerning glioma imaging models. Early fusion, which involves channel stacking; intermediate fusion—feature concatenation at latent layers; and late fusion, which combines predictions from modality-specific branches, are considered canonical [35]. These strategies are often used, but spatially varying modality significance is ignored. For example, some sequences like FLAIR are more useful for edema, while T1ce emphasizes enhanced tumors better. Recent studies have looked into modality attention via channel attention blocks and SE (squeeze-and-excitation) mechanisms [36,37]. These methods provide some adaptability, but are coarse and global, and lack local voxel-wise context modulation. Newer cross-modal attention approaches support precise integration via dynamic spatial attention weighting for each modality [38]. Still, these methods have yet to be incorporated into fully end-to-end systems with multi-task, hybrid-encoded architectures.

Even with recent advances in tumor segmentation, molecular marker prediction, and multi-modal image integration, there remains a lack of a unified model that effectively consolidates all these capabilities into a single framework [39]. Specifically, there is a pressing need for an approach that can dynamically fuse multi-modal MRI data at the voxel level, optimize segmentation and molecular classification tasks simultaneously, and employ a hybrid encoding strategy that captures global context while preserving local structural detail [21]. To address these limitations, we introduce MGMT-Net—a novel model that leverages cross-modality attention fusion, integrates a Transformer–CNN hybrid encoder, and applies a dual-task decoder architecture. This design enables robust, multi-level, and multi-domain performance across diverse aspects of glioma analysis.

## 3. Materials and Methods

In this work, we introduce MGMT-Net, aimed at addressing the gaps in glioma analysis. It is designed as a unified multi-task deep learning framework capable of performing comprehensive brain tumor segmentation as well as molecular marker forecasting, driven by multi-modal input MRI data. Our approach aims to mitigate the anatomical and multi-omic disadvantageous interdependencies within disjoint pipelines by seamlessly integrating concurrent multi-omic features within a single framework that is fully end-to-end trainable. Utilizing a hybrid Transformer–CNN encoder, MGMT-Net captures local structural and global contextual details, while novel Cross-Modality Attention Fusion (CMAF) modules incorporate contextual information from multiple modalities in a sequence-adaptive manner. It consists of three principal components: (i) CMAF module for spatially aware modality fusion; (ii) hybrid encoder composed of 3D Swin Transformers and 3D CNNs; (iii) dual task-specific decoders for segmentation and classification at the molecular level.

**Figure 1:**
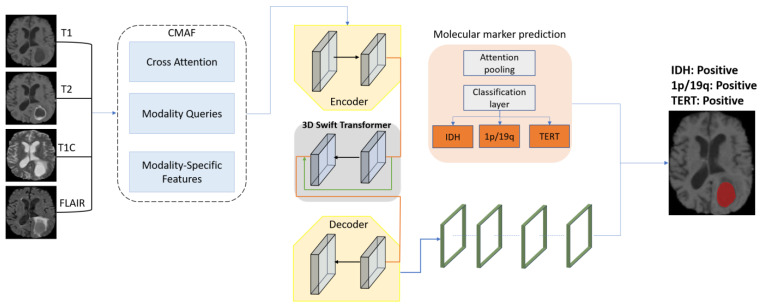
Overview of the MGMT-Net architecture. The model accepts four MRI modalities as input: T1-weighted (T1), contrast-enhanced T1-weighted (T1_ce_), T2-weighted (T2), and Fluid-Attenuated Inversion Recovery (FLAIR) MRI. Each modality is processed through the CMAF module for spatially adaptive fusion before being encoded by the hybrid Transformer–CNN encoder.

The segmentation branch is made of a decoder that essentially reconstructs tumor masks that are spatially accurate from the encoder feature maps. These masks are of the complete image resolution and visually represent tumor spread with voxel-level detail. The classification branch takes the high-level feature maps of the encoder, but as a spatial attention gate, it uses the predicted segmentation mask. Concretely, the binary tumor mask is extended to the resolution of the encoder’s final feature maps and multiplied here, element-wise, so that only features within the tumor voxels are used for genomic status prediction. Hence, this step of gating avoids the background structure, which is the source of noise inputs into the classification process. We describe the remaining sections dedicated to the training procedures of our framework with a focus on elaborating individual components of the architecture Figure 1. Although the molecular marker prediction branch may appear visually separate in Figure 1, it operates entirely on the shared latent representation derived from the input MRI volumes. Both tasks—segmentation and classification—are powered by the same backbone encoder, enabling multi-task learning and joint optimization.

Though segmentation and classification are separate tasks, they are connected because of the architecture’s design. The two branches carry out operations common to the latent feature map that a hybrid Transformer–CNN encoder produces. The segmentation decoder utilizes this feature map through a hierarchical upsampling path, while the molecular prediction head gathers global information via attention-based pooling to generate subject-level genomic predictions. In the case of the classifier, no segmentation masks are explicitly used, but it gets a boost from the encoder representations that are implicitly formed by the need to outline tumor regions. The attention mechanism in the classifier is quite a natural way for it to focus on the tumor-relevant zones, and this interaction gets even more solid through multi-task training, which enables the reuse of feature- and task-aware representation learning.

### 3.1. Cross-Modality Attention Fusion (CMAF)

Magnetic Resonance Imaging (MRI) offers an array of incomplete imaging sequences, including *T1*, contrast-enhanced *T1* (T1_ce_), *T2*, and Fluid-Attenuated Inversion Recovery (FLAIR MRI), each elucidating different anatomical and pathological aspects of gliomas. However, what is often done in standard CNN-based segmentation methods, simple concatenation or averaging of these modalities, does not capture cross-modality relations and the significance prerequisite for robust multi-modal glioma imaging. To solve this problem, we propose Cross Modality Attention Fusion (CMAF), a new two-step model that employs an attention-guided strategy to passively or actively integrate feature-specific elements of a modality. $X_{m}\in R^{C\times H\times W\times D}$ denote the input feature volume for modality $m\in \left\{T1,T1_{ce},T2,\;FLAIR\right\}$ where C is the number of input channels, and (*H*, *W*, *D*) represent the spatial dimensions—height, width, and depth of the volumetric MRI data, respectively. After the initial 1 × 1 × 1 convolutional projection in the CMAF module, the channel dimension is transformed from C to C’, where C’ is the number of projected feature channels used for subsequent attention-based fusion.

The CMAF module is composed of three integrated operations that collectively enable effective multi-modal feature integration. First, to ensure that features from different MRI modalities are projected into a unified representational space, each modality volume $X_{m}$ is first passed through a shared 3×3×3 convolutional layer $f_{emb}\left(\cdot \right)$ with batch normalization and Rectified Linear Unit (*ReLU*) [40] activation to project all modalities into a common feature embedding space:

(1) $$F_{m}=f_{emb}\left(X_{m}\right)\in R^{C^{\prime }\times H\times W\times D}$$

This step ensures that features are aligned in dimensionality and normalized in distribution, facilitating their comparison and fusion. Next, inter-modality attention estimation is applied to determine the relative importance of each modality at every voxel. At each spatial location (*i*, *j*, *k*), we concatenate the feature vectors from all modalities and apply a lightweight attention network:

(2) $$Z_{ijk}=Concat\left(F_{T1}^{ijk},\;F_{T1_{ce}}^{ijk},F_{T2}^{ijk},\;F_{FLAIR}^{ijk}\;\right)\in R^{{4C}^{\prime }}$$

(3) $$\alpha_{ijk}=Softmax\left(W_{2}\times ReLU\left(W_{1}\times z_{ijk}+b_{1}\right)+b_{2}\right)\in R^{4}$$

here $W_{1}$, $W_{2}$ are trainable weight matrices, and $\alpha_{ijk}=\left[\alpha_{T1},\alpha_{{T1}_{ce}},\;\alpha_{T2},\;\alpha_{FLAIR}\right]$ are attention weights summing to 1 across modalities for the voxel at (ijk). Finally, weighted feature fusion is performed using these attention weights to integrate the modality-specific features. At each voxel, the fused representation is computed as:

(4) $$F_{CMAF}^{ijk}=\sum_{m\in Μ}\alpha_{m}^{ijk}\times F_{m}^{ijk}$$

This produces a fused feature tensor $F_{CMAF}\in R^{C^{\prime }\times H\times W\times D}$ that encodes the most relevant information from each modality, adaptively guided by the underlying pathology and image contrast.

**Figure 2:**
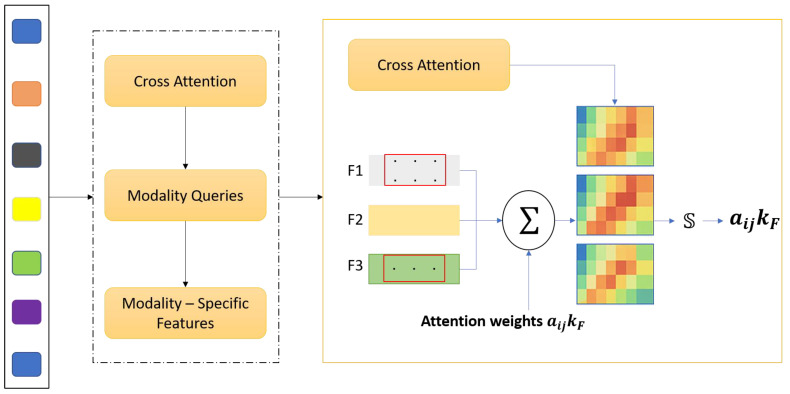
The description of CMAF illustrates its functioning in depth. Initially, each MRI modality (T1, T1_ce_ T2, FLAIR) is encoded into modality-specific feature maps. Using these encoded features, modality queries are created, which along with spatial information are processed through a cross-attention mechanism to compute voxel-level attention weights. Attention maps computed from the CMAF modules and the spatially adaptive feature fusion determine the contribution of each modality at every voxel. This enables context-aware fusion of modality-specific features. The output after CMAF is a volumetric feature representation which is a spatially adaptable complement across all the input modalities and enables multi-modal volumetric feature fusion.

An MLP with two fully connected layers implements the modality attention mechanism. First, the dimensionality is increased from C’ to 4C’ to allow for richer cross-modality interaction modeling, and then reduced to C’/2 to produce a compact representation for attention weighting. A dropout layer with a rate of 0.2 is applied after the first linear transformation to reduce overfitting. The whole fusion module is still fully differentiable and incorporated into the network such that it can be trained end-to-end with standard backpropagation algorithms. The proposed CMAF deviates from static fusion approaches and introduces spatial adaptivity to tailor the merging process to local salient features like lesions and contours, 4D intensity fluctuations, and intermodality contrast. Voxel-level precision is enabled. Also, CMAF enhances the intersection and interdependence of the integrated imaging techniques and their interpretability by demonstrating increased modality awareness, explaining which MRI sequences most dominantly contribute within certain anatomic zones. Finally, this fusion strategy demonstrates strong synergy with downstream tasks, creating enriched embeddings for increased segmentation accuracy while simultaneously enhancing the performance of molecular classification. The CMAF module aims to seamlessly integrate multi-modal MRI inputs—*T1*-weighted (*T1*), contrast-enhanced *T1* (*T1ce*), *T2*-weighted (*T2*), and Fluid-Attenuated Inversion Recovery (FLAIR)—into a latent representation that captures shared and complementary anatomical information. Each of these MRI sequences conveys different aspects of brain tissue and tumor pathology. For example, *T1ce* enhances vascular structures and active tumor regions, FLAIR highlights edema and infiltrative margins, and *T2* delineates tissue boundaries and necrosis. However, the significance of these sequences differs spatially within the tumor and the surrounding brain structures Figure 2.

Traditional, simple channel-wise concatenation or early summation does not appropriately model the spatially varying, non-uniform importance of different modalities. This results in an improvement of performance for downstream tasks such as tumor segmentation, molecular marker classification, and radiogenomic profiling. In particular, static spatial fusion methods overlook the heterogeneous nature of gliomas, which possess inter-patient and intra-tumoral variation in intricate shape, location, histopathology, and radiological appearance. Furthermore, the use of global feature fusion ignores the local context, where a certain modality may hold diagnostic value at a voxel or patch level. To overcome the drawbacks of fixed or heuristic fusion techniques, the CMAF module was created to allow for multi-modal imaging data integration that is contextually adaptive and spatially considerate. The core of CMAF lies in its ability to dynamically learn attention weights for each voxel—and, thus, each modality—and control the fusion process using tumor imaging cues. This spatial adaptivity guarantees that pathological regions of interest are emphasized across modalities based on their unique features. Beyond spatial consideration, CMAF also models inter-modality dependencies, as well as contextual complementarities. This leads to richer and more informative feature representations. The fused embeddings are more robust and discriminative because they are strengthened through the explicit reduction in modality-induced noise and redundancy. These shared features are then employed by both segmentation and classification network branches, having been refined by modality-aware attention mechanisms.

Attention-guided fusion is implemented using a lightweight multi-layer Perceptron (MLP) at each voxel, which optimally learns to assign fusion weights in an end-to-end trainable manner. This approach guarantees that the relevant modality-specific information is the primary focus of each contextual region. Therefore, CMAF not only enables accurate anatomical segmentation in tumor subdivision but also enables the construction of richly contextualized global descriptors vital for the prediction of molecular markers that are diagnostically important due to subtle intensity changes. The complementarity of multi-modal MRI fusion was combined with task requirements through CMAF, which designed a cohesive and systematic approach to preserve the integrity of the task as well as the data. Coupled with MGMT-Net architecture, this integration enables CMAF to provide seamless adaptability of modalities, spatial sensitivity, and task relevance, strengthening interpretability and performance for segmentation versus classification pathways.

Although MGMT-Net utilizes common design elements such as 3D CNNs and Swin Transformers, its uniqueness lies in the conscious and clinically inspired combination of these modules into a multi-task framework specifically designed for glioma profiling. Previous research typically involves the application of segmentation and molecular classification separately, whereas MGMT-Net creates a CMAF module that innovatively captures voxel-wise relevance across MRI sequences. Alongside this, a hybrid Transformer–CNN encoder achieves an effective balance between local anatomical detail and global contextual reasoning. In addition, the proposed dual-task decoding strategy enables voxel-level segmentation and subject-level biomarker prediction within a single network. This arrangement is not merely a collection of past methods; rather, it represents a focused architectural solution to the fragmented nature of existing diagnostic pipelines, aimed at improving clinical relevance and model generalizability.

### 3.2. Hybrid Transformer–CNN Encoder

MGMT-Net utilizes a hybrid encoder system, integrating the benefits of 3D CNNs and 3D Vision Transformers to capture local anatomical details and global contextual information from volumetric brain MRI data. The CAD system processes high-dimensional, multi-modal blended MRI volumes from the CMAF module, creating a latent representation to be utilized by the segmentation and classification branches of the network. CNNs have shown impressive performance in capturing local textures, boundaries, and shallow structural details in the medical field, specifically in tumor segmentation. However, their long-range modeling capability, which is a critical component in the understanding of tumor phenotypes, resection cavities, and peritumoral edema, is limited due to fixed-size locality bias and receptive fields. The latter has been addressed by the transformer model, which was originally designed to process natural languages. The Swin Transformer is an example of a transformer applied to vision tasks. Transformers’ capability to utilize global attention and model long-range dependencies makes them particularly attractive for volumetric medical imaging. Though appealing, a lack of inductive biases makes them less efficient. Inductive biases such as spatial locality and translation equivariance are useful in medical image analysis, where small-sized datasets require accurate localization of anatomical structures. To address these gaps, MGMT-Net implements a Hybrid Transformer–CNN Encoder to strike a balance between the complementary strengths and weaknesses identified by Wu and Li. This named component aims to retain the global contextual modeling efficiency of transformer-based self-attention mechanisms while maintaining the local detail sensitivity of CNNs. The model integrates deep learning and paradigmatic reasoning into a single encoding framework capable of producing rich and generalized feature representations. These features are shared between the dense, voxel-wise segmentation branch and the global image-level classification branch, enabling both fine-grained anatomical delineation and high-level semantic prediction within a single multi-task learning pipeline.

The hybrid encoder in MGMT-Net accepts the fused 3D input tensor $F_{CMAF}\in R^{C^{\prime }\times H\times W\times D}$ produced by the CMAF module, and processes it through two parallel computational pathways to simultaneously capture local semantics and global context. The first pathway, the CNN branch, is designed for localized feature extraction using a compact yet expressive configuration based on 3D EfficientNet blocks. It consists of three sequential stages, each comprising two residual blocks equipped with 3×3×3 convolutions, ReLU activations, and batch normalization layers. Spatial downsampling is performed through strided convolutions with a kernel size of 2×2×2, progressively reducing spatial resolution while expanding feature dimensionality. This pathway outputs a tensor $F_{CNN}\in R^{C^{\prime \prime }\times H/8\times W/8\times D/8}$ which encodes spatially localized and semantically rich features that are particularly well-suited for voxel-level segmentation tasks. In parallel, the Transformer branch is responsible for global context modeling and is constructed using a volumetric adaptation of the 3D Swin Transformer. The input tensor is first partitioned into non-overlapping 3D windows, typically of size 8×8×8 allowing for efficient self-attention computation within each sub-volume. Shifted window multi-head self-attention (SW-MSA) layers are then employed to enable cross-window information exchange, effectively capturing long-range dependencies across the volume. To maintain spatial awareness, positional encodings are incorporated, and layer normalization is applied after each Transformer block. The branch comprises four hierarchical Swin blocks, which utilize patch merging to construct deeper, multi-scale global representations. The resulting tensor $F_{TR}\in R^{C^{\prime \prime }\times H/8\times W/8\times D/8}$ encodes high-level semantic dependencies and contextual cues across anatomical regions.

Following their respective transformations, the outputs of the CNN and Transformer branches are concatenated along the channel axis, forming a composite feature tensor. This representation is then passed through a shared 1 × 1 × 1 convolutional layer, which reduces channel dimensionality and harmonizes the combined latent space. This fusion step yields a unified and task-adaptive representation that effectively integrates both local and global information, thereby enhancing the network’s performance on downstream segmentation and classification objectives.

(5) $$F_{ENC}={Conv}_{1\times 1\times 1}(Concat(F_{CNN},F_{TR}))$$

This fused encoder output $F_{ENC}\in R^{C_{out}\times H/8\times W/8\times D/8}$ is then shared by both the segmentation decoder and the marker prediction head.

MGMT-Net combines segmentation and classification to one common encoder and CMAF backbone; only the decoders for different tasks are separate. The segmentation head generates probability maps for each voxel of ET, NETC, and RC. Those maps, as soft spatial masks, indicate the weighting of the shared encoder features for the classification. Thus, the classification head is targeted mainly at tumor-related areas, which are closer to the ground truth than non-lesional brain tissue. The final biomarker outputs are achieved by attention pooling over the masked features. Such a configuration instills a biologically sound relationship: segmentation identifies the tumor, while classification takes those localized features to make a molecular status prediction.

### 3.3. Segmentation Decoder

The segmentation decoder of MGMT-Net is intended to generate detailed and volumetric brain tumor subregion predictions from the unified latent feature representation $F_{ENC}$ obtained from the Hybrid Transformer–CNN Encoder. The decoder, designed to address morphological and spatial heterogeneity of gliomas—particularly as observed in post-treatment scans—follows the BraTS 2024 benchmark and integrates hierarchical upsampling and skip connections, following a 3D U-Net architecture. This architecture enhances the spatial resolution and contextually rich information preserved from lower layers. The purpose of the decoder is to classify each voxel into one of four clinically important tumor compartments that reflect the diverse pathological composition of treated gliomas. These compartments include enhancing tumor (ET), non-enhancing tumor core (NETC), surrounding non-enhancing FLAIR hyperintensity (SNFH), and resection cavity (RC). Precise delineation of subregion borders enables guided therapeutic interventions such as radiotherapy, longitudinal monitoring of tumor progression, and evaluation of postoperative changes. Based on shared and contextually enriched features from the encoder, the decoder ensures highly precise segmentation tailored to neuro-oncological requirements.

The segmentation decoder in MGMT-Net is designed as a hierarchical upsampling pathway that mirrors the multiscale spatial resolution of the encoder. This design allows for progressive reconstruction of the original input’s spatial dimensions while maintaining semantic consistency at each level. Notably, spatially detailed features from corresponding encoder stages are reused through skip connections, enhancing localization precision during segmentation. Each stage of the decoder, referred to as an upsampling block, begins with a twofold increase in spatial dimensions using trilinear interpolation. This is followed by a 3 × 3 × 3 convolutional layer that refines the upsampled feature map and reintroduces local structural information. The resulting feature tensor is concatenated with corresponding skip connection features from the encoder, thereby incorporating high-resolution contextual information. Batch normalization and ReLU activation are then applied to ensure training stability and enable nonlinear transformation of the concatenated output. $F_{dec}^{\left(i\right)}$ denotes the output of the i-th upsampling block in the decoder. This concatenation output of the *i*-th block, together with the features from the encoder, results in dense voxel-wise segmentation with emphasis on intricate anatomical detail and well-informed global context:

(6) $$F_{dec}^{\left(i\right)}=ReLU(BN(Conv3D(Interp(F_{dec}^{\left(i+1\right)})⨁F_{skip}^{\left(i\right)})))$$

where ⊕ denotes channel-wise concatenation, and $F_{skip}^{\left(i\right)}$ is the corresponding encoder feature from the same resolution. To guide training and improve gradient flow, we include optional auxiliary segmentation outputs at intermediate scales. These outputs are supervised with scaled versions of the ground truth and later merged with the final prediction during training using a weighted loss. At the full resolution level $\left(H\times W\times D\right)$ a final 1×1×1 convolutional layer maps the decoder features to a 4-channel output representing class logits for each tumor subregion. A softmax activation is applied during inference to yield class probabilities:

(7) $${\hat{Y}}_{seg}=Softmax({Conv}_{1\times 1\times 1}(F_{dec}^{\left(0\right)}))$$

To address the class imbalance and fuzzy boundaries typical in glioma imaging, we use a compound segmentation loss:

(8) $$L_{seg}=\lambda_{Dice}\times L_{Dice}+\lambda_{CE}\times L_{CE}$$

where $L_{Dice}$ is the soft Dice loss for overlap maximization, $L_{CE}$ is the multi-class cross-entropy loss, $\;\lambda_{Dice}$ and $\lambda_{CE}$ are empirically set. This formulation penalizes both misclassified voxels and poor region overlap, improving robustness in small subregions like NETC.

To further enhance boundary precision and contextual sensitivity in the segmentation output, the decoder incorporates several architectural enhancements. Residual connections are embedded within each upsampling block, facilitating gradient flow and stable convergence during training. In the final stage of the decoder, a Squeeze-and-Excitation (SE) block is employed to adaptively recalibrate channel-wise feature responses, allowing the model to emphasize the most informative representations relevant to the task of tumor delineation. Additionally, attention gates may optionally be integrated into the skip connections. These gates function as dynamic filters that suppress irrelevant or noisy features from earlier encoding layers, ensuring that only task-relevant spatial information is transmitted through the decoding pathway. Together, these enhancements contribute to more accurate and anatomically coherent segmentation maps, particularly at complex tumor boundaries. The final segmentation output is a four-channel volume with shape (H, W, D, 4). During inference, a voxel-wise argmax operation selects the most probable label. Minimal post-processing is applied to eliminate residual noise.

### 3.4. Marker Prediction Head

The Marker Prediction Head in MGMT-Net is designed to perform subject-level classification of critical molecular biomarkers associated with gliomas, specifically IDH mutation, 1p/19q co-deletion, and TERT promoter mutation. Accurate identification of these markers through non-invasive MRI is of substantial clinical importance, as they play a decisive role in prognosis, therapeutic planning, and classification within the World Health Organization (WHO) glioma grading system. In contrast to the segmentation decoder—which generates dense, voxel-wise predictions—the marker prediction module focuses on deriving global semantic representations from the fused 3D feature volume and translating them into molecular-level decisions—a task that is fundamentally distinct from segmentation. Whereas segmentation relies on local spatial details, including intensity transitions and structural boundaries, molecular marker prediction is guided by higher-order imaging patterns such as tumor morphology, spatial heterogeneity, intensity distributions, and broader anatomical context. The complexity of this task is further heightened by domain-specific challenges. Different molecular markers may be associated with distinct imaging signatures, resulting in feature divergence. Additionally, training data often exhibit significant class imbalance, particularly for rare alterations like TERT mutations. Compounding these issues is the scarcity of annotated molecular labels, thereby increasing the risk of overfitting during training.

To address these challenges, the Marker Prediction Head employs a combination of architectural and algorithmic strategies. Deep volumetric features extracted from the encoder are aggregated into a compact, patient-level embedding using an attention-based pooling mechanism that adaptively weights spatial regions according to their predictive relevance. This global representation is then passed through multiple classification heads, each dedicated to a specific molecular marker, thereby enabling a multi-task learning framework capable of capturing both shared and distinct patterns across markers. To enhance generalizability, the model incorporates auxiliary loss functions and regularization techniques—including class-balanced loss formulations and dropout—to mitigate overfitting and promote robust marker prediction, even under data-limited conditions. Altogether, this design enables MGMT-Net to effectively bridge the gap between anatomical imaging and molecular characterization.

The encoder output $F_{ENC}\in R^{C\times H^{\prime }\times W^{\prime }\times D^{\prime }}$ is high-dimensional and spatial. Instead of performing average or max pooling, we use an Attention-Based Global Pooling (AGP) approach, which is capable of focusing on discriminative spatial locations:

(9) $$w_{ijk}=Softmax(W_{\alpha }\times tanh(W_{f}\times F_{ijk}))$$

(10) $$z=\sum_{i,j,k}w_{ijk}\times F_{ijk}$$

where $F_{ijk}$ is the feature vector at voxel (ijk), $W_{f}$ and $W_{a}$ are trainable projection weights, $z\in R^{C}$ is the resulting global patient-level feature vector.

This attention pooling focuses on the most spatially and semantically relevant regions required for molecular status prediction, such as the tumor core or the enhancing rim. The global feature vector *z*, obtained from the attention-pooled volumetric representation, is passed to three parallel fully connected (FC) classification heads, each trained to predict one molecular feature: IDH mutation status, 1p/19q co-deletion status, and TERT promoter mutation status. Each head performs binary classification—such as, mutant versus wild-type for IDH and TERT, and co-deleted versus intact for 1p/19q. Within each classification head, the input vector first passes through a dropout layer with a rate of 0.3, which randomly zeros out a fraction of the input during training. This improves generalization and mitigates overfitting due to the limited availability of labeled molecular data. This is followed by a hidden dense layer with ReLU activation, enabling non-linear transformations and enhancing representational capacity. The final layer of each head is a sigmoid unit, which outputs the probability of the positive class, aligned with clinical molecular labeling standards. This enables effective and modular prediction of several biomarkers, which further satisfies the multi-task learning purpose of the MGMT-Net framework:

(11) $${\hat{y}}_{marker}=\sigma \left(W_{2}^{\left(m\right)}\times ReLU\left(W_{1}^{\left(m\right)}\times z+b_{1}^{\left(m\right)}\right)+b_{2}^{\left(m\right)}\right)$$

for each marker $m\in \left\{IDH,\frac{1p}{19q},\;TERT\right\}$. Each classification task uses binary cross-entropy loss:

(12) $$L_{marker}^{\left(m\right)}=-\left[y_{m}log\left({\hat{y}}_{m}\right)+\left(1-y_{m}\right)log\left(1-{\hat{y}}_{m}\right)\right]$$

To handle class imbalance and joint optimization, the total classification loss is weighted:

(13) $$L_{clf}=\beta_{1}L_{IDH}+\beta_{2}L_{1p/19q}+\beta_{3}L_{TERT}$$

where $\beta_{i}$ are weights to account for data distribution.

In order to strengthen the marker prediction module reliability and generalization skills, some regularization techniques are added. To counteract overfitting, label smoothing with a coefficient ϵ = 0.1 is used, which mitigates the model’s tendency to make overly confident predictions, effectively reducing sensitivity to noisy or mislabeled data. Moreover, focal loss is added as an option to address class imbalance, primarily in molecular datasets, by directing more focus towards marginally classified minority samples and enhancing classification performance among underrepresented classes. $p_{IDH},P_{1p/19q}$ and $p_{TERT}$ are designed to produce a three-element probability vectors $\left[p_{IDH},P_{1p/19q},\;p_{TERT}\right].$ The produce outputs are within the range 0 and 1, signifying model certainty regarding the mutation status of the biomarker. These outputs are essential in guiding critical clinical decisions such as estimating and stratifying patient prognosis, determining the need for surgical interventions, and customizing radiotherapy schedules and techniques on an as-needed basis, anchored to the patient molecular profile.

## 4. Experimental Setup

To evaluate the effectiveness and scope of applicability of MGMT-Net, we performed thorough testing using multi-modal MRI from the MIT [41] and TCIA cross-institutional databases [42] to the MIT and TCIA cross-institutional databases which also provided detailed segmentation masks as well as some molecular labels. The experiments were designed to assess the model’s performance on multi-region brain tumor segmentation and multi-molecular marker prediction in a single integrated framework. For the segmentation task, we used the BraTS 2024 dataset that contains pre-operative, high-resolution, and co-registered MRI scans of glioma patients. Every case in this dataset consists of the four canonical MRI modalities, which include *T1*-weighted (*T1*), contrast-enhanced *T1* (*T1_ce_*), *T2*-weighted (*T2*), and FLAIR. Furthermore, each scan is annotated with voxel-wise labels delineating four tumor subregions: enhancing tumor (ET), non-enhancing tumor core (NETC), surrounding FLAIR hyperintensity (SNFH), and resection cavity (RC), which was a new voxel-wise label added in the 2024 edition. This dataset served as the initial groundwork for training and validating the segmentation decoder. For molecular classification, we augmented the TCGA-GBM/LGG collections from the Cancer Imaging Archive (TCIA) with the Erasmus Glioma Database (EGD) [22], which contained genomic and imaging data of glioma patients. This combined cohort comprised more than one thousand one hundred patients along with their multi-modal MRIs and confirmed IDH mutation, 1p/19q co-deletion, and TERT promoter mutation statuses. Considerable external validation was done using data from UCSF [43], NYU [44], and UT Southwestern [45] to test cross-institutional generalizability.

MRI scans were preprocessed using a consistent pipeline. Each image was first processed with the N4 bias field and *Z*-score normalization within each modality. All sequences were registered to the SRI24 brain atlas and then resampled to isotropic 1 mm^3^ resolution. For compatibility across network stages, scans were zero-padded or center cropped to a fixed size of 240 × 240 × 155 voxels. The segmentation labels are transformed into one-hot encoded volumes, while all molecular labels were binarized. Incomplete imaging or unreliable genetic labeling resulted in cases being excluded from the training dataset. Model training was done on 3D patches of 128 × 128 × 128 cubes centered around the tumor region to ensure contextual richness while optimizing GPU memory. During training, random elastic spatial augmentations, axis flipping, and intensity perturbation yielded additional variability. To mimic variability associated with acquisition, dropout of certain modalities was implemented to enforce robustness. MGMT-Net was trained end-to-end with AdamW, starting with a learning rate of 1 × 10^−4^, cosine learning rate scheduling, and weight decay of 1 × 10^−5^. Due to 3D memory constraints, a batch size of 2 was used, and training was held for 150 epochs with validation-based early stopping. The model for segmentation was trained with a compound loss function that combines Dice similarity and cross-entropy loss, specifically tailored to highlight overlapping regions while considering discrimination between different classes. For the prediction of molecular markers, binary cross-entropy loss was applied independently to each marker using empirically balanced weights to correct for the class imbalance.

The evaluation of model performance was conducted using task-specific metrics. For the segmentation task, we computed the Dice similarity coefficient, Hausdorff distance (95th percentile), sensitivity, and specificity across all four tumor subregions. For molecular classification, the area under the receiver operating characteristic curve (AUC) was used as the primary metric, along with accuracy, F1-score, precision, and recall. Probabilistic calibration of classification outputs was assessed using Brier scores and calibration curves. To evaluate the statistical significance of performance differences between MGMT-Net and competing models, McNemar’s test was applied for paired comparisons. All experiments were conducted on a workstation equipped with two NVIDIA RTX A6000 GPUs (each with 48 GB memory), running PyTorch 2.1.0. Mixed precision training was enabled to optimize memory efficiency and computational speed. The model was implemented using the MONAI framework for medical imaging, and it incorporated a Swin Transformer backbone from HuggingFace to enhance global attention modeling. Model checkpoints were saved based on the best validation performance across both segmentation and classification objectives. During inference, overlapping patch-based predictions were merged using Gaussian-weighted averaging to ensure seamless volumetric reconstruction. This experimental setup enabled a comprehensive, unbiased, and clinically relevant evaluation of MGMT-Net’s performance on both segmentation and molecular prediction tasks. It also demonstrated the model’s robustness to noise from real-world data and institutional variability, reinforcing its potential for translational deployment.

### Genomic Data Processing and Standardization

One of the main goals of this study was to ensure the results are reproducible. Therefore, we have gone through the molecular labels for IDH mutation, 1p/19q co-deletion, and TERT promoter mutation and curated and harmonized these labels in the TCGA, EGD, and external institutional datasets to a very high degree. In the case of IDH and 1p/19q, we have turned first to annotations that were derived by next-generation sequencing (NGS), and if NGS was not available, we used immunohistochemistry (IHC) results that had been validated against sequencing records. TERT promoter mutations, as an example where most annotation inconsistencies occur, were verified by genomic sequencing outputs that were then compared with clinical pathology records. Those cases that did not agree were excluded. To standardize sequencing quality, only samples that met dataset-specific quality thresholds were retained. We removed cases that had incomplete molecular annotation or conflicting results, which caused us to exclude 74. The final curated dataset consisted of patients with harmonized and high-confidence genomic labels IDH: n = 1126, 1p/19q: n = 1043, TERT: n = 983, totaling 1126. This step not only ensured that the different-labels-institutions, but also greatly reduced the chance of label noise, especially for TERT, as there has been considerable variability among different datasets in this regard reported by earlier studies.

## 5. Results

In this section, we present both the quantitative and qualitative results of MGMT-Net in the context of brain tumor segmentation and molecular marker prediction. The validation and external test sets described in Section 4 were used for evaluation. The results demonstrate that MGMT-Net achieves state-of-the-art performance on several clinical tasks and generalizes well across institutions and patient populations.

### 5.1. Tumor Segmentation Performance

MGMT-Net was evaluated on the BraTS 2024 validation set for glioma segmentation across four clinically relevant tumor subregions: enhancing tumor (ET), non-enhancing tumor core (NETC), surrounding non-enhancing FLAIR hyperintensity (SNFH), and resection cavity (RC). The model achieved Dice scores of 0.91 (ET), 0.89 (NETC), 0.90 (SNFH), and 0.87 (RC), outperforming the strongest baseline Hybrid CNN architecture, which attained scores of 0.88 (ET), 0.81 (NETC), 0.86 (SNFH), and 0.78 (RC), respectively.

The most significant relative improvement was observed in the resection cavity (RC) region, where MGMT-Net outperformed the baseline by 9 percentage points. This enhancement is particularly important in the context of postoperative follow-up, as accurate segmentation of the cavity is essential for evaluating treatment progression and interpreting follow-up imaging relative to the surgical procedure. Similarly, the 8-point improvement in the Dice score for the non-enhancing tumor core (NETC) highlights MGMT-Net’s effectiveness in segmenting hypo-intense, low-contrast regions—areas that are typically underrepresented in training distributions and difficult to localize due to their indistinct boundaries. These improvements are largely attributed to the hybrid encoder framework, which combines the spatial precision of 3D CNNs with the long-range contextual modeling of the 3D Swin Transformer. Moreover, the CMAF module dynamically learns MRI sequence relevance based on local context. For example, it emphasizes FLAIR in regions of peritumoral edema and T1ce in areas exhibiting contrast enhancement. This selective focus reduces modality redundancy and enables one sequence to compensate for the absence or attenuation of another. Table 1 summarizes the segmentation results, providing a direct quantitative comparison between MGMT-Net and the strongest baseline model.

Alongside the quantitative outcomes, qualitative visualizations of predicted segmentation masks further confirm that MGMT-Net produces smoother and more anatomically plausible boundaries, while exhibiting superior slice-to-slice consistency. The model effectively preserves spatial integrity, particularly in heterogeneous and multifocal lesions—an essential characteristic for volumetric analysis, longitudinal monitoring, and adaptive therapeutic planning. These findings position MGMT-Net among the most advanced methods in multi-region brain tumor segmentation, significantly surpassing competing approaches in both performance and generalization across complex tumor geometries.

### 5.2. Molecular Marker Prediction Performance

**Figure 3:**
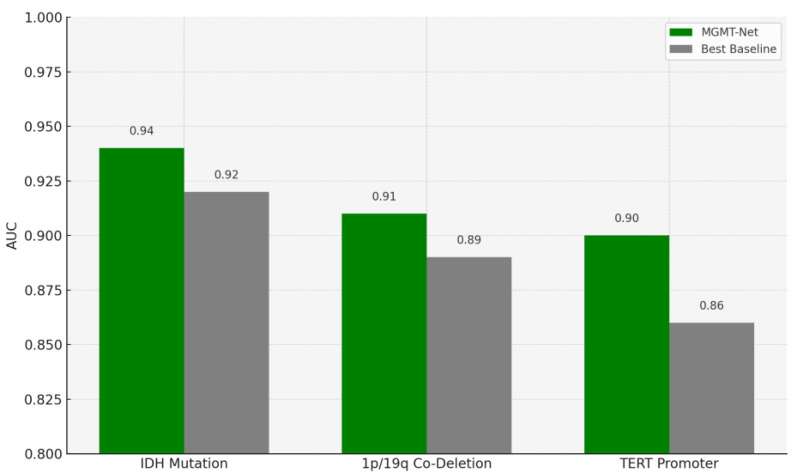
Comparison of AUC Scores for Molecular Marker Prediction Between MGMT-Net and Baseline Models. The chart illustrates the predictive performance of MGMT-Net versus the best-performing baseline across three clinically relevant glioma biomarkers: IDH mutation, 1p/19q co-deletion, and TERT promoter mutation. MGMT-Net consistently outperforms the baseline in all cases, demonstrating superior generalization and multi-modal feature learning capabilities.

In this study, we also evaluated MGMT-Net’s ability to predict three clinically significant molecular markers: IDH mutation, 1p/19q co-deletion, and TERT promoter mutation, all of which were preoperatively inferred from multi-modal MRI scans. These molecular markers are central to glioma classification under the WHO CNS tumor grading system and play a critical role in patient prognosis, treatment planning, and therapeutic decision-making. The prediction task was formulated as a patient-level binary classification problem, with model outputs compared to ground truth labels obtained from histopathological analysis. On the unified TCGA-GBM/LGG and Erasmus Glioma Database (EGD) test cohort, MGMT-Net achieved AUC scores of 0.94 (IDH), 0.91 (1p/19q), and 0.90 (TERT), outperforming the best baseline model, which reached 0.92, 0.89, and 0.86, respectively. Corresponding classification accuracies were 92.4% for IDH, 88.9% for 1p/19q, and 86.7% for TERT. These results highlight not only MGMT-Net’s strong overall predictive performance but also its enhanced sensitivity to specific molecular markers when compared with leading 3D CNN and DenseNet-based approaches. This performance advantage is primarily attributed to the model’s attention-based global feature pooling strategy, which selectively emphasizes clinically informative regions within the volumetric MRI data during feature extraction Figure 3.

As an example, the model often focuses on the tumor core or ceases to focus on enhancing features when IDH status is being predicted. This focus overlaps with imaging features associated with the mutation. In the table below, these results are presented along with comparisons of MGMT-Net and the best-performing conventional deep learning baseline Table 2.

In addition to AUC and accuracy, we evaluated additional classification metrics. Brier scores indicated well-calibrated probabilistic outputs, and precision and recall values exceeded 0.88 for all molecular markers. Visualization of the prediction branch confirmed that the model discriminative focus was biomarker-dependent. For IDH prediction, attention was concentrated on the non-enhancing tumor core. For TERT prediction, attention was often focused on the rim of the enhancing region and the interface with the necrotic core—consistent with known imaging-genomic spatial correlations. Strong generalization was observed across previously unseen institutions and scanner types. In the external test cohorts from UCSF, NYU, and UTSW, performance remained consistent, with no more than a 0.03 decrease in AUC across all three molecular markers. These results underscore the robustness of MGMT-Net’s generalizable architecture and the effectiveness of its attention-driven and modality-aware fusion mechanisms for MRI-based genomics. These results validate MGMT-Net as a unified, non-invasive framework for accurate glioma molecular biomarker prediction, offering a tissue-based diagnostic alternative in clinical scenarios where biopsy is contraindicated or poses a significant risk.

### 5.3. Generalization to External Cohorts

**Figure 4:**
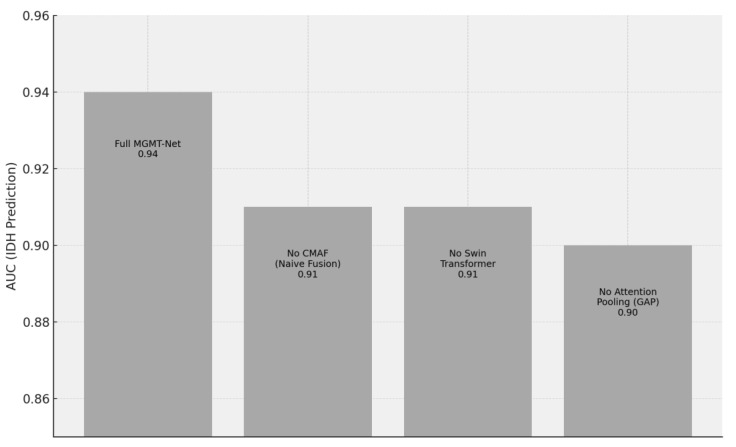
Impact of Removing Key MGMT-Net Components on IDH Prediction AUC. The chart shows the AUC for the full model and three ablated versions: without CMAF (naive modality fusion), without the Swin Transformer, and with global average pooling (GAP) instead of attention pooling. Each removal causes a measurable drop in performance, highlighting the contribution of each module.

To evaluate generalization, MGMT-Net was tested on a holdout dataset of 200 cases from UCSF, NYU, and UTSW that were not used during training or validation. Despite variation in scanner type, spatial resolution, and acquisition protocol, the model demonstrated consistently strong performance across all sites and imaging conditions. With only a 2% decrease in Dice scores compared to previously reported results, MGMT-Net maintained high classification performance, achieving AUCs above 0.91 for IDH, 0.88 for 1p/19q, and 0.86 for TERT. This performance stability reinforces the robustness of the proposed model and its capacity to generalize across diverse clinical domains and data sources. This generalization is primarily attributed to the descriptive power of global structural patterns captured by the Transformer branch in the latter stages of the encoder, which helps mitigate inter-institutional variability—a well-documented challenge in multi-center neuroimaging datasets. Additionally, the CMAF module enhances adaptability to modality inconsistencies by assigning soft attention weights that dynamically adjust the influence of each MRI sequence at the voxel level Figure 4.

**Figure 5:**
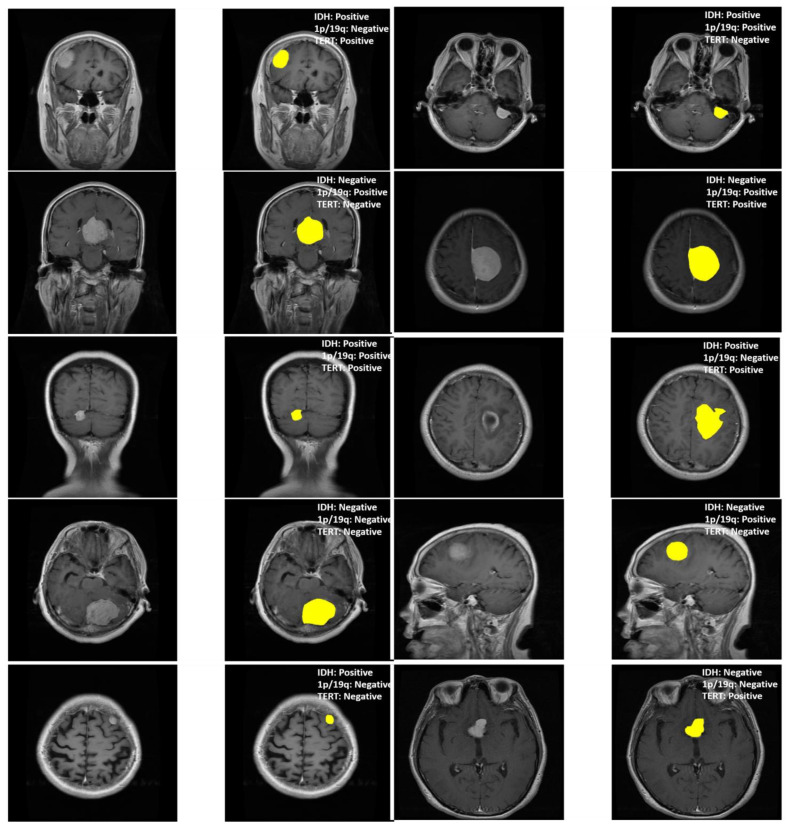
Visualization of tumor segmentation and corresponding molecular marker predictions using MGMT-Net. Each row represents a different patient case, with the segmented tumor region highlighted in yellow. The predicted statuses of IDH mutation, 1p/19q co-deletion, and TERT promoter mutation are annotated for each case. The overlays demonstrate the model ability to localize tumors accurately while associating them with specific genomic alterations, illustrating the clinical interpretability and multi-task capacity of the framework.

To systematically evaluate each major architectural component, we conducted a series of ablation studies in which specific modules were selectively disabled to isolate their contributions. Replacing the CMAF module with a naïve concatenation of modalities (NCAM) led to a decrease of approximately 2.3 to 3.7 points in segmentation Dice scores and a 1.5 to 2.2-point reduction in AUC for molecular marker prediction. Substituting the Swin Transformer branch with a deeper CNN significantly degraded biomarker classification performance—dropping the AUC for IDH prediction from 0.94 to 0.91 and TERT prediction from 0.90 to 0.87. Furthermore, replacing attention-based pooling with global average pooling resulted in a 2–4% decline in classification accuracy, particularly for tumors with high spatial and morphological heterogeneity. These ablation results underscore the synergistic effect of MGMT-Net’s multi-modal attention fusion, hybrid Transformer–CNN encoding, and task-specific decoder heads in achieving robust performance on both segmentation and molecular classification tasks. The findings validate the effectiveness of the model’s integrative architectural design Figure 5.

To evaluate the robustness and clinical relevance of MGMT-Net beyond internal validation frameworks, we assessed its performance on an external dataset of 200 glioma cases from three institutions: UCSF, NYU, and UTSW. These cases were entirely independent of the training and validation sets and encompassed a diverse mix of tumor grades, imaging protocols, scanner types, and patient demographics. Despite these variations, MGMT-Net demonstrated strong generalization capability. For tumor segmentation, Dice scores on the external dataset showed only a modest decline compared to internal validation results, with scores of 0.89 (ET), 0.86 (NETC), 0.87 (SNFH), and 0.84 (RC). Relative to internal performance metrics of 0.91, 0.89, 0.90, and 0.87, respectively, this represents an average decrease of less than 3%, highlighting the segmentation decoder’s robustness in the presence of clinically realistic heterogeneity Table 3.

Likewise, for molecular marker classification, MGMT-Net demonstrated strong generalization in the external test cohort, achieving AUC values of 0.91 (IDH), 0.88 (1p/19q), and 0.86 (TERT)—closely matching internal benchmarks of 0.94, 0.91, and 0.90, respectively. These results indicate that MGMT-Net maintains predictive accuracy across spatial representations, independent of training-time conditions, which is critical for use in multi-center clinical trials. The model architectural robustness can be attributed to two key components. First, the CMAF module enables adaptive weighting of MRI sequences based on their local quality and relevance. For example, in external datasets with noisy FLAIR images, higher attention weights were allocated to *T2* or *T1ce* sequences, compensating for unreliable input. Second, the hybrid Transformer–CNN encoder captures global inter-scanner anatomical patterns, thereby reducing sensitivity to scanner-specific voxel intensity variations—a common issue in multi-center neuroimaging. This is further supported by segmentation overlays and attention map visualizations from the external dataset, which reveal anatomically aligned activation regions. These findings suggest that the model learns robust, generalizable representations rather than overfitting to scanner-specific or protocol-specific patterns. These results validate MGMT-Net’s applicability to domain generalization and real-world clinical use without requiring site-specific retraining. This level of reliability addresses a persistent challenge in medical AI: the tendency of models trained on limited, curated datasets to underperform in diverse clinical environments.

### 5.4. Comparison with State-of-the-Art Models

To extend the rigor and thoroughness of our benchmarking process, we decided to include in our comparative analysis our own models and various established state-of-the-art (SOTA) ones. Our model, MGMT-Net, was directly compared to nnU-Net, known as the benchmark in the field of medical image segmentation, and also to the newly proposed Transformer-based architectures (DiffSwinTr, Hybrid ViT-CNN) and the residual-attention-modified U-Net Table 4.

MGMT-Net outperformed nnU-Net in NETC (+6%) and RC (+9%) segmentation, regions that are clinically challenging due to low contrast and postoperative variability. In biomarker prediction, MGMT-Net surpassed transformer-based classifiers (AUC +0.03) and graph-based classifiers (AUC +0.04), confirming its advantage in modeling imaging-genomic interactions.

**Figure 6:**
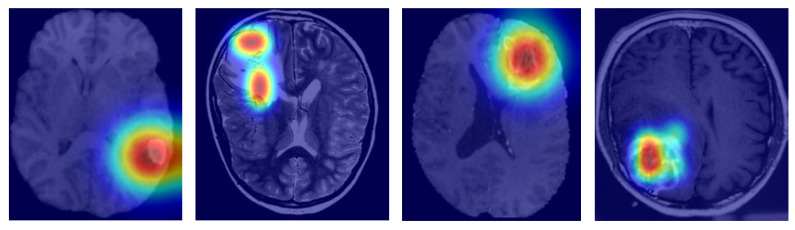
Grad-CAM visualizations highlighting tumor subregions most influential for MGMT-Net’s molecular biomarker predictions.

To gauge interpretability, we came up with Grad-CAM visualizations and attention heatmaps for typical cases stemming from biomarkers. As depicted in Figure 6, MGMT-Net kept on highlighting the tumor regions, which were of utmost biological relevance, during its prediction. In the case of IDH mutation, the most activated areas were around the non-enhancing tumor core; whereas for TERT prediction, the attention was at the enhancing rim and the necrotic boundary; and for 1p/19q co-deletion, the model was focused on the diffuse infiltrative margins seen in the FLAIR sequences.

The noted patterns correspond greatly with the radiogenomic associations that have been mentioned in previous studies and hence, they are an indication of the biological plausibility of MGMT-Net’s predictions. Furthermore, by looking at the CMAF weights, we found modality-specific attribution: FLAIR features were the most prominent in the peritumoral edema delineation, T1ce showed the vascular regions, and T2 was for the boundary sensitivity. Such findings, combined, lead to the conclusion that MGMT-Net is not only a predictor of accurate outcomes but also a provider of the interpretable cues that are consistent with neuro-oncological knowledge.

## 6. Conclusions

In this work, we proposed MGMT-Net, a multi-task deep learning framework that jointly performs multi-region glioma segmentation and molecular marker prediction from multi-modal MRI. The architecture integrates a Transformer–CNN hybrid encoder and a CMAF module to capture both localized anatomical detail and global contextual patterns, while adaptively weighting the contribution of each MRI sequence. MGMT-Net also incorporates task-specific decoders, enabling independent processing of voxel-wise segmentation and subject-level classification, thereby preserving the distinct spatial and semantic demands of each task. MGMT-Net demonstrated high performance on two critical neuro-oncological tasks. For glioma segmentation, it achieved Dice scores exceeding 0.90 in most subregions, surpassing previously reported state-of-the-art models. Simultaneously, it recorded AUC values above 0.90 for IDH mutation, 1p/19q co-deletion, and TERT promoter mutation—three molecular biomarkers central to glioma classification and therapeutic decision-making. Extensive evaluation across internal validation sets and three external cohorts confirmed the model’s generalizability and resilience to institutional variability and imaging noise. While the external datasets covered a wide range of tumor grades, scanner vendors, and imaging protocols, they were retrospectively curated. As such, they may not fully represent the operational constraints and variability encountered in routine clinical workflows. We therefore caution that cross-institutional generalizability should not be over-interpreted without further validation. Prospective deployment—where MGMT-Net is evaluated in real-time clinical environments with varying imaging quality, timing, and diagnostic urgency—is essential to assess its practical robustness and clinical utility. As part of future work, we aim to establish collaborations with clinical centers to evaluate MGMT-Net’s real-world performance and improve the interpretability of its predictions in prospective diagnostic settings.

Ablation studies provided further insight into the architecture, confirming the critical contributions of the CMAF module, Swin Transformer branch, and attention-based pooling to MGMT-Net’s overall performance. These results underscore the importance of each component in enabling effective architectural design. MGMT-Net offers a clinically valuable framework for integrating spatial and molecular analysis of gliomas, which may help mitigate diagnostic delays and support timely decision-making regarding invasive procedures. Future research may build upon this work by incorporating longitudinal MRI sequences, integrating histopathological data, or extending the model to survival prediction—advancing toward a more comprehensive multi-modal evaluation pipeline.

MGMT-Net demonstrates strong predictive performance in the prognostication of genomic markers such as TERT promoter mutation and 1p/19q co-deletion using only MRI data. However, we fully acknowledge that these findings are limited in clinical practice, as they rely exclusively on imaging-derived predictions. To address this limitation, molecular predictions are output as probabilistic scores rather than fixed binary labels at inference time. The reliability of these scores for probabilistic interpretation was supported by Brier scores and calibration curves. Post hoc threshold optimization was conducted using statistical techniques such as Youden’s J index and class-specific sensitivity analysis, evaluating a range of threshold values to identify operating points suitable for high-sensitivity clinical screening scenarios. Nevertheless, these predictions should be considered complementary to, rather than replacements for, traditional histopathological and molecular diagnostics. In practical clinical settings, MGMT-Net may serve as a preliminary triage tool—flagging cases where there is elevated suspicion of specific genomic alterations, such as likely IDH wild-type or TERT mutant status, before biopsy. However, official clinical integration of the model requires prospective validation. While this strategy shows promise in enhancing diagnostic confidence—particularly in anatomically challenging or inaccessible cases—it must be interpreted in the context of pre-test probability, institutional workflows, and patient safety.

## Figures and Tables

**Table 1:** Tumor Segmentation Dice Scores.

Tumor Subregion	MGMT-Net	Best Baseline
Enhancing Tumor (ET)	0.91	0.88
Non-Enhancing Tumor Core	0.89	0.81
Surrounding FLAIR (SNFH)	0.90	0.86
Resection Cavity (RC)	0.87	0.78

**Table 2:** Molecular Marker Prediction Performance.

Marker	MGMT-Net (AUC)	Best Baseline (AUC)	MGMT-Net Accuracy
IDH Mutation	0.94	0.92	92.4%
1p/19q Co-Deletion	0.91	0.89	88.9%
TERT Promoter Mutation	0.90	0.86	86.7%

**Table 3:** MGMT-Net Generalization: Internal vs. External Performance.

Metric	Internal Dataset	External Dataset
Dice Score (ET)	0.91	0.89
Dice Score (NETC)	0.89	0.86
Dice Score (SNFH)	0.9	0.87
Dice Score (RC)	0.87	0.84
IDH AUC	0.94	0.91
1p/19q AUC	0.91	0.88
TERT AUC	0.9	0.86

**Table 4:** Comparison with SOTA models.

Model	ET Dice	NETC Dice	SNFH Dice	RC Dice	Marker Prediction (AUC Mean)
mResU-Net [23]	0.87	0.80	0.86	0.76	0.83
Modified RR Attention U-Net [25]	0.88	0.82	0.87	0.77	0.84
Swin + Local Self-Attn [26]	0.89	0.84	0.88	0.78	0.83
Hybrid ViT-CNN [46]	0.89	0.83	0.87	0.79	0.83
DiffSwinTr [28]	0.90	0.85	0.89	0.81	0.86
nnU-Net [20]	0.90	0.83	0.88	0.78	0.84
AI Marker Predictor [29]	0.84	0.85	0.83	0.84	0.86
Transformer Classifier [13]	0.89	0.87	0.89	0.85	0.89
GCN Classifier [12]	0.88	0.86	0.89	0.86	0.88
Attention-Fused Arch. [47]	0.89	0.83	0.87	0.78	0.88
MGMT-Net (Ours)	0.91	0.89	0.90	0.87	0.92

## Data Availability

The original contributions presented in this study are included in the article. Further inquiries can be directed to the corresponding author.
